# Internet Addiction, Smartphone Addiction, and Hikikomori Trait in Japanese Young Adult: Social Isolation and Social Network

**DOI:** 10.3389/fpsyt.2019.00455

**Published:** 2019-07-10

**Authors:** Masaru Tateno, Alan R. Teo, Wataru Ukai, Junichiro Kanazawa, Ryoko Katsuki, Hiroaki Kubo, Takahiro A. Kato

**Affiliations:** ^1^Tokiwa Child Development Center, Tokiwa Hospital, Sapporo, Japan; ^2^Department of Neuropsychiatry, Sapporo Medical University, School of Medicine, Sapporo, Japan; ^3^VA Portland Health Care System, HSR&D Center to Improve Veteran Involvement in Care, Portland, OR, United States; ^4^Department of Psychiatry, Oregon Health & Science University, Portland, OR, United States; ^5^School of Public Health, Oregon Health & Science University and Portland State University, Portland, OR, United States; ^6^School of Psychological Science, Health Sciences University of Hokkaido, Tobetsu-cho, Japan; ^7^Department of Neuropsychiatry, Graduate School of Medical Sciences, Kyushu University, Fukuoka, Japan

**Keywords:** internet addiction, smartphone addiction, behavioral addiction, hikikomori, social withdrawal

## Abstract

**Background:** As the number of internet users increases, problems related to internet overuse are becoming more and more serious. Adolescents and youth may be particularly attracted to and preoccupied with various online activities. In this study, we investigated the relationship of internet addiction, smartphone addiction, and the risk of hikikomori, severe social withdrawal, in Japanese young adult.

**Methods:** The subjects were 478 college/university students in Japan. They were requested to complete the study questionnaire, which consisted of questions about demographics, internet use, the Internet Addiction Test (IAT), the Smartphone Addiction Scale (SAS)–Short Version (SV), the 25-item Hikikomori Questionnaire (HQ-25), etc. We investigated the difference and correlation of the results between two groups based on the purpose of internet use or the total score of each self-rating scale, such as screened positive or negative for the risk of internet addiction, smartphone addiction, or hikikomori.

**Results:** There was a trend that males favored gaming in their internet use while females used the internet mainly for social networking *via* smartphone, and the mean SAS-SV score was higher in females. Two-group comparisons between gamers and social media users, according to the main purpose of internet use, showed that gamers used the internet longer and had significantly higher mean IAT and HQ-25 scores. Regarding hikikomori trait, the subjects at high risk for hikikomori on HQ-25 had longer internet usage time and higher scores on both IAT and SAS-SV. Correlation analyses revealed that HQ-25 and IAT scores had a relatively strong relationship, although HQ-25 and SAS-SV had a moderately weak one.

**Discussion:** Internet technology has changed our daily lives dramatically and altered the way we communicate as well. As social media applications are becoming more popular, users are connected more tightly to the internet and their time spent with others in the real world continues to decrease. Males often isolate themselves from the social community in order to engage in online gaming while females use the internet as to not be excluded from their communications online. Mental health providers should be aware of the seriousness of internet addictions and hikikomori.

## Introduction

The number of internet users in Japan has exceeded 100 million and continues to grow. Internet technology has changed our daily lives dramatically. Adolescents and youth may particularly be attracted to and preoccupied with various online activities. A nationwide survey conducted by the Ministry of Internal Affairs and Communications of Japan (MIAC) in 2017 demonstrated that 96.9% of teenagers in Japan use the internet on a daily basis (1).

As the number of internet users increases, problematic internet use, or internet addiction, has been rising as well. The term internet addiction has been used frequently and casually to describe individuals with severe internet overuse or problematic internet use (2–5). Kimberly Young, an American psychologist, initially proposed provisional diagnostic criteria for subjects with problematic internet use with her naming of “internet addiction” in reference to *Diagnostic and Statistical Manual of Mental Disorders* (DSM) IV (6) criteria for substance dependence in 1996 (7). Two years later, Young revised her definition of internet addiction, which made it more similar to an impulse control disorder in DSM-IV (8). It has been controversial whether internet addiction is a clinical entity in psychiatry since Young’s proposal about two decades ago (5, 9–11). Internet addiction has not been listed as a psychiatric disorder in the DSM-5 in 2013 (12) nor the International Classification of Diseases (ICD)-11, which was released in June 2018 (13). However, after thorough discussion, some regard it as one type of behavioral addiction and this attitude seems to be the majority (3, 14–16).

In recent years, internet access has been changing. The results of the MIAC survey (n = 38,630) reported that 59.7% of users access the internet through a smartphone, while 52.5% of them use the internet *via* desktop and/or laptop computers (1). Dominance of the smartphone is more prominent in younger age groups compared to senior age groups. A lot of teens have their smartphones with them throughout the day due to smartphones having superb mobility and multifunction capability. They can connect to the internet anytime and anywhere.

This phenomena of a rapid increase of smartphone users can be observed throughout the world. With more people using smartphones, problems regarding smartphone overuse become more serious. On these backgrounds, the concept of smartphone addiction has attracted the attentions of researchers from several countries (17–22), while debate about whether it is one of the behavioral addictions or not has been lasting (23).

Our previous study demonstrated that male internet users favored online gaming while female users were online for social networking services (SNSs) *via* their smartphones (24–26). The results from a national survey conducted by MIAC in 2013 also came to a similar conclusion; in that male users preferred playing games on the internet while female users mostly used it for communications with their friends (27). It is notable that 68.4% of teenagers use SNSs, according to the MIAC survey (1). It appears that adolescents and youth maintain their friendship primarily by communicating on the internet, especially young women.

Hikikomori phenomena, severe social withdrawal, is one of the most serious social concerns in Japan over the last two decades (28, 29). The concept of hikikomori was first introduced to the public when a Japanese psychiatrist, Tamaki Saito, published a book in 1998 with the name in its title (30). In his book *Social Withdrawal (Shakaiteki Hikikomori)*, Saito defined hikikomori provisionally as “those who withdraw entirely from society and stay in their own homes for more than six months, with onset usually during the latter half of their twenties, and for whom other psychiatric disorders do not better explain the primary causes of this condition.” Since then, hikikomori has attracted the interest of researchers and a few epidemiological studies have been carried out in Japan. In 2006, the World Mental Health Japan Survey reported that the prevalence of hikikomori was approximately 1.2% of the Japanese population and 232,000 families had ongoing hikikomori subjects (31).

In response to social needs for development of effective interventions to hikikomori, Japan’s Ministry of Health, Labor and Welfare (MHLW) published a guideline for hikikomori in 2010 (32). In this guideline, hikikomori is defined as “a situation where a person without psychosis is withdrawn into his/her home for more than six months and does not participate in society such as attending school and/or work.” In accordance with this definition, several studies have been conducted, including well-structured multinational studies (33–37), and social interest in hikikomori phenomena has been growing around the world.

Japanese government pays much attention to hikikomori and regards this phenomenon to be of high importance. The Cabinet Office of the Japanese government published results of their study on lifestyle of the youth in 2016 and reported that the number of hikikomori was estimated to be 176,000 in a narrow sense (core hikikomori) and 541,000 in a broad sense (the total of both core hikikomori and on the verge of hikikomori) (38). In the definition of this report, pre-hikikomori condition means people who spend most of the time in their own room or home but go out for shopping at convenience stores near their houses. In contrast with the original concept of hikikomori, the recent concept of hikikomori allows the subjects with hikikomori to avoid going out but still have social contact with others over the internet (39). To prevent development of severe hikikomori, early detection and early intervention to pre-hikikomori is an important issue of the society.

Gradually, hikikomori phenomena gained attention from both Japanese and foreign researchers, as well as several academic papers that were published even outside of Japan (40–50). Recently, most of hikikomori subjects are using internet in common while they are shutting themselves in their own homes. Some papers are suggesting the close relationship between internet addiction and hikikomori (43, 49).

In this study, we investigated the relationship between internet use and hikikomori trait in Japanese young adults.

## Methods

Data presented in this study were collected as part of a multicenter study for the development of effective interventions to hikikomori, a severe social withdrawal syndrome, which is directed by one of the co-authors of this paper (TK).

### Subjects

The subjects of this study were 487 college and university students in Sapporo and its environs, Japan. All colleges and universities are private, and their deviation scores are average or a little below the average. Research collaborators for data collection were recruited through personal connections of the first author of this paper (MT). Nine teachers from three universities and six colleges agreed to participate in this study voluntarily and distributed questionnaire sheets as a printed matter in their classrooms. In this study, the subjects received the questionnaire sheets and filled out the questionnaire completely in the classroom.

### Study Questionnaire

The study questionnaire consisted of questions about demographics (age, gender, etc.), internet use (length of internet use on weekdays and weekends, purpose of internet use, favorite SNS, etc.), Young’s Internet Addiction Test (IAT) (51), the Smartphone Addiction Scale (SAS)–Short Version (SV) (17, 18), the 25-item Hikikomori Questionnaire (HQ-25) (52), and Tarumi’s modern-type depression trait scale; Avoidance of social roles, Complaint and low Self-esteem (TACS) (53). The results of TACS were excluded from statistical analyses for this paper because validity and reliability of this scale were not yet confirmed at the time of writing this paper.

It took about 15 to 20 min for respondents to complete the questionnaire. A research collaborator at each study site was watching and waiting in the classroom to support respondents upon their requests.

### Smartphone Addiction Scale–Short Version

The original version of the SAS was developed as a self-report scale in South Korea (18). The standard SAS includes 33 questions that assess six factors relating to smartphone overuse on a six-point Likert scale ranging from 1 (strongly disagree) to 6 (strongly agree). Kwon et al. also developed the short version of SAS (SAS-SV) by extracting 10 questions from standard SAS to use it as a smartphone addiction screener in adolescents (17). Medical professionals, including the developer of SAS, confirmed the validity of the SAS-SV by having an interview with randomly selected subjects in South Korea. In translating the SAS-SV into Japanese, there was a little modification by inserting the term LINE (54) into the sentence of Q8 in the standard SAS: “Constantly checking my smartphone so as not to miss conversations between other people on Twitter or Facebook.”

SAS and/or SAS-SV has been translated into Chinese, Malay, Arabic, Brazilian-Portuguese, Spanish, and French (55–59). The reliability and validity of the translated version of SAS have been examined in several countries. An investigation of the reliability and validity of the Japanese version of the SAS and SAS-SV has been in progress (60).

### Young’s Internet Addiction Test

Young’s IAT has 20 items regarding internet overuse. All items begin with the phrase “How often do you…”, e.g., “How often do you try to cut down the amount of time you spend online and fail?” Respondents are requested to choose one of the following scores: 5 = always, 4 = often, 3 = frequently, 2 = occasionally, and 1 = rarely. The IAT has been used to measure the severity of internet addiction. The total score of IAT ranges from 20 to 100. In the present study, we classified the level of internet addiction according to the cutoff points previously reported by Young (51) and by referring to previous studies by Japanese researchers (61–63). In this study, the total IAT scores for each group were less than 40 points for the average online user, 40 to 69 for possibly addicted to the internet, and 70 to 100 points for severe internet addiction. The reliability and validity of the Japanese version of the IAT have been investigated (64, 65).

### The 25-Item Hikikomori Questionnaire

The HQ-25 was developed as a self-administered instrument for assessing severity of symptoms of hikikomori over the preceding 6 months (52). The HQ-25 consisted of 25 questions regarding psychological features and behavioral patterns of typical hikikomori syndrome, such as lack of social connectedness, active social isolation or withdrawal behavior, avoidance of social contact, and a sense of alienation from society. All items of HQ-25 were rated on a scale ranging from 0 (strongly disagree) to 4 (strongly agree). Among 25 items, 6 items need to be reverse-scored to create a total scale score. The HQ-25 has a score range of 0–100. Developers of HQ-25 proposed a cutoff score of 42 (out of 100), which was associated with a sensitivity of 94% and specificity of 61% in their clinical study.

### Statistical Analysis

Questionnaire sheets with blank answer(s) were excluded from statistical analyses. As a result, 24 out of 511 collected sheets were excluded.

Statistical analyses were completed using StatFlex Ver.6. Welch’s t-test was used to compare the mean of two groups. Pearson’s correlation coefficient was calculated to investigate the association between two factors. In all cases, statistical significance was set at p < 0.05.

### Ethics

This study was approved by the ethics committee of Specified Medical Corporation Sapporo Yushin-no-sato Tokiwa Hospital. The study’s aim was stated on the cover page of the questionnaire sheets that requested voluntary respondents to answer all questions anonymously. Answering the questions was deemed to constitute consent.

## Results

There were 487 respondents (132 male, 355 female) who completed study questionnaires. The mean age was 19.6 ± 1.5 (males 20.2 ± 1.8/females 19.4 ± 1.4) years with an age range between 18 and 28. Summary of the results are shown in **Table 1**.

**Table 1:** Summary of the results.

	Whole	Male	Female	p value
	**(n = 487)**	**(n = 132)**	**(n = 355)**
Age	19.6 ± 1.5	20.2 ± 1.8*	19.4 ± 1.4	*p < 0.0001
Internet use (h)				
Weekdays	4.86 ± 3.1	4.80 ± 3.2	4.89 ± 3.1	p = 0.7947
Weekend	6.82 ± 4.1	6.71 ± 4.2	6.86 ± 4.0	p = 0.7182
Purpose (%)				
Gaming	42 (8.6)	25 (18.9)	17 (4.8)	
SNS	303 (62.2)	54 (40.9)	249 (70.1)	
Video-sharing	101 (20.7)	38 (28.8)	63 (17.8)	
Music	23 (4.7)	9 (6.8)	14 (3.9)	
Web searches	13 (2.7)	4 (3.0)	9 (2.5)	
Others	5 (1.0)	2 (1.5)	3 (0.9)	
Favorite SNS (%)				
LINE	286 (58.7)	88 (66.7)	198 (55.8)	
Twitter	70 (14.4)	23 (17.4)	47 (13.2)	
Facebook	6 (1.2)	2 (1.5)	4 (1.1)	
Instagram	117 (24.0)	13 (9.9)	104 (29.4)	
Others	8 (1.7)	6 (4.5)	2 (0.6)	
SAS-SV	29.6 ± 8.8	27.4 ± 8.2	30.4 ± 8.9*	*p = 0.0005
Above cutoff	194 (39.3)	48 (36.4)	146 (41.1)	
IAT	41.0 ± 13.1	41.8 ± 15.5	40.7 ± 12.2	p = 0.4729
<40	255 (52.4)	66 (50.0)	189 (53.2)	
40≤ IAT <70	216 (44.4)	58 (43.9)	158 (44.5)	
70≤	16 (3.3)	8 (6.0)	8 (2.3)	
HQ-25	28.1 ± 16.3	29.5 ± 16.9	27.5 ± 16.1	p = 0.2552
<42	379 (77.8)	103 (78.0)	276 (77.8)	
42≤	108 (22.2)	29 (22.0)	79 (22.3)	

*p < 0.05.

### Internet Use

The mean length of internet use was 4.86 ± 3.1 (males 4.80 ± 3.2/females 4.89 ± 3.1) h on weekdays and 6.82 ± 4.1 (males 6.71 ± 4.2/females 6.86 ± 4.0) h on weekends without statistically significant differences in gender. The most common purpose of internet use was SNS in both genders (40.9% among males, 70.1% among females, 62.2% among all subjects), followed by video-sharing and gaming. The rate of internet use for gaming was higher among males (18.9%) than females (4.8%).

Participants were also asked what SNS they preferred out of LINE, Twitter, Facebook, Instagram, and others. LINE was selected as the most popular SNS application by both male and female groups (66.7% among males, 55.8% among females, 58.7% among all subjects) followed by Twitter among males (17.4%) and Instagram among females (29.4%).

### Smartphone Addiction Scale–Short Version

The overall SAS-SV scores were 29.6 ± 8.8 (the total score distributed from 10 to 59) for all subjects, 27.4 ± 8.2 for males, and 30.4 ± 8.9 for females. Female subjects had a significantly higher SAS-SV score than males. This finding was consistent with results from previous studies in South Korea (17, 18). Kwon and his group proposed gender-specific SAS-SV cutoff points for males (a cutoff value of 31) and females (a cutoff value of 33). According to the proposed cutoff points, 48 out of 132 male subjects (36.4%) screened positive and 146 out of 355 female subjects (41.1%) screened positive as well.

### Young’s Internet Addiction Test

Concerning the IAT, the overall score was 41.0 ± 13.1 (the total score distributed from 0 to 86), with 255 respondents (52.4%) regarded as the average online users (IAT <40), 216 (44.4%) regarded as possible addiction (IAT 40 to 69), and 16 (3.3%) regarded as severe addiction (IAT 70 and higher). Referring to previous studies that classified subjects with 70 or higher on the IAT as severe internet addiction (51), 3.3% of our subjects were IA. Mean IAT scores and the number of subjects (%) in respective score ranges on IAT (average online uses, possibly addicted and severely addicted to the internet) in each gender were as follows: 41.8 ± 15.5, 66 (50.0%), 58 (43.9%), and 8 (6.0%), respectively, among males; and 40.7 ± 12.2, 189 (53.2%), 158 (44.5%), and 8 (2.3%), respectively, among females. Statistical analyses revealed non-significant difference in the mean IAT score.

### The 25-Item Hikikomori Questionnaire

The overall HQ-25 scores were 28.1 ± 16.3 (the total score distributed from 0 to 82) for all subjects, 29.5 ± 16.9 for males, and 27.5 ± 16.1 for females. There was no difference in the HQ-25 scores between males and females (Welch’s t-test, p = 0.2552). Based on the cutoff score proposed by the developer of HQ-25, in the male group, 29 out of 132 subjects (22.0%) had scores indicating being at risk for hikikomori.

### Two Group Comparison between Gamers and SNS Users

We analyzed the results of this study by making two groups according to the main purpose of internet use, gaming, and SNS. Table 2 shows the summary of the results. Regarding the mean length of internet use in both weekdays and weekends, mean IAT score, and mean HQ-25 score, all of them were significantly longer and higher in the subjects who used the internet primarily for gaming. The only result that did not show significance was the mean SAS-SV score.

**Table 2:** A two-group comparison between gamers and SNS users.

	Gaming	SNS	p value
	**(n = 42)**	**(n = 303)**
Age	20.1 ± 1.4*	19.4 ± 1.4	*p = 0.0043
Internet use (h)			
Weekdays	6.40 ± 4.1*	4.85 ± 3.1	*p = 0.0211
Weekend	8.86 ± 5.2*	6.70 ± 3.9	*p = 0.0136
SAS-SV	32.1 ± 7.9	30.1 ± 8.8	p = 0.1317
Above cutoff	23 (54.8)	118 (38.9)	
IAT	49.6 ± 15.8*	39.7 ± 11.9	*p = 0.0003
<40	12 (28.6)	173 (57.1)	
40≤ IAT <70	25 (59.5)	124 (40.9)	
70≤	5 (11.9)	6 (2.0)	
HQ-25	36.3 ± 17.8*	24.7 ± 14.2	*p = 0.0002
<42	27 (64.3)	257 (84.8)	
42≤	15 (35.7)	46 (15.2)	

*p < 0.05.

The two group comparison between male gamers (n = 25) and male SNS users (n = 54) demonstrated that male gamers used the internet significantly longer in the weekend (9.00 ± 5.0 vs 6.30 ± 4.1, p = 0.00256) and scored significantly higher on IAT (48.2 ± 18.1 vs 38.7 ± 12.9, p = 0.0245), SAS-SV (30.6 ± 8.1 vs 26.2 ± 8.1, p = 0.0285), and HQ-25 (32.9 ± 17.8 vs 21.9 ± 11.5, p = 0.0082), compared to male SNS users, although there was not a significant difference in the length of internet use in weekdays (6.32 ± 3.8 vs 4.61 ± 3.2, p = 0.0593).

### Hikikomori Trait and Internet Addiction

Two-group comparisons were performed between the subjects who scored 42 and higher on HQ-25 (at high risk for hikikomori) and those with an HQ-25 score of less than 42 (low hikikomori-trait). The results are shown in Table 3. Subjects with high risk for hikikomori scored significantly higher on both SAS-SV and IAT, and used the internet longer than subjects with low risk for hikikomori.

**Table 3:** A two-group comparison between subjects at high and low risk for hikikomori.

	At high risk for hikikomori	At low risk for hikikomori	p value
	**(n = 108)**	**(n = 379)**
Age	19.8 ± 1.9	19.5 ± 1.4	p = 0.0681
Internet use (h)			
Weekdays	5.48 ± 3.7*	4.66 ± 2.9	*p = 0.0298
Weekend	7.71 ± 4.4*	6.53 ± 3.9	*p = 0.0091
SAS-SV	31.2 ± 9.2*	29.1 ± 8.6	*p = 0.0302
Above cutoff	54 (50.0)	140 (36.9)	
IAT	47.6 ± 14.5*	38.9 ± 11.7	*p < 0.0001
<40	30 (27.8)	225 (59.4)	
40≤ IAT <70	69 (63.9)	147 (38.8)	
70≤	9 (8.3)	7 (1.9)	

*p < 0.05.

### Correlation among HQ-25, SAS-SV, and IAT

**Figure 1:**
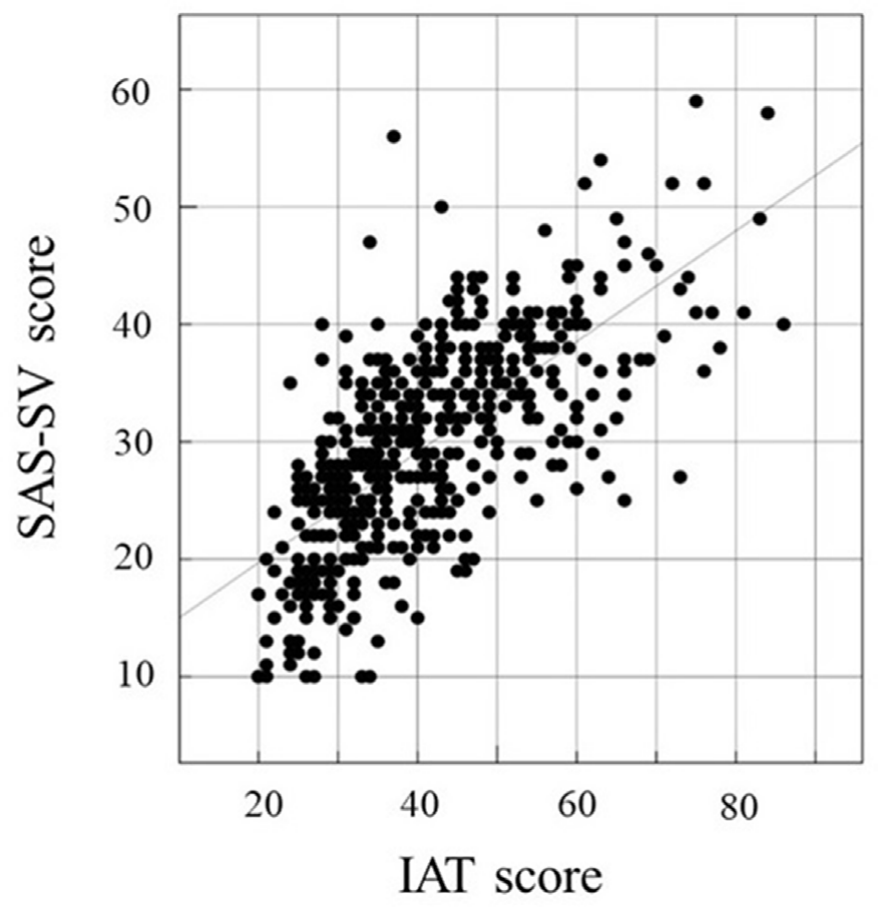
Correlation of Internet Addiction Test (IAT) (51) score and Smartphone Addiction Scale–Short Version (SAS-SV) (17) score. The correlation coefficient was 0.6931 (95% conﬁdence interval, 0.6439 − 0.7366, p < 0.00001).

**Figure 2:**
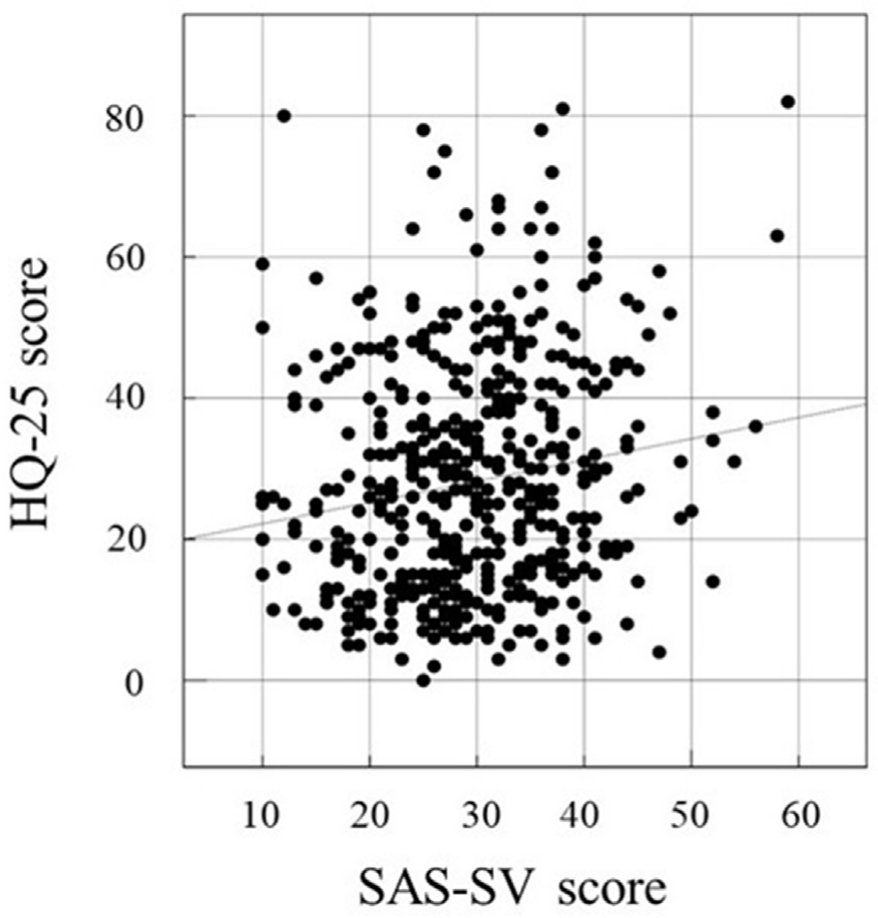
Correlation of SAS-SV (17) score and 25-item Hikikomori Questionnaire (HQ-25) (52). The correlation coefficient was 0.1636 (95% conﬁdence interval, 0.0758 − 0.2488, p = 0.00029).

**Figure 3:**
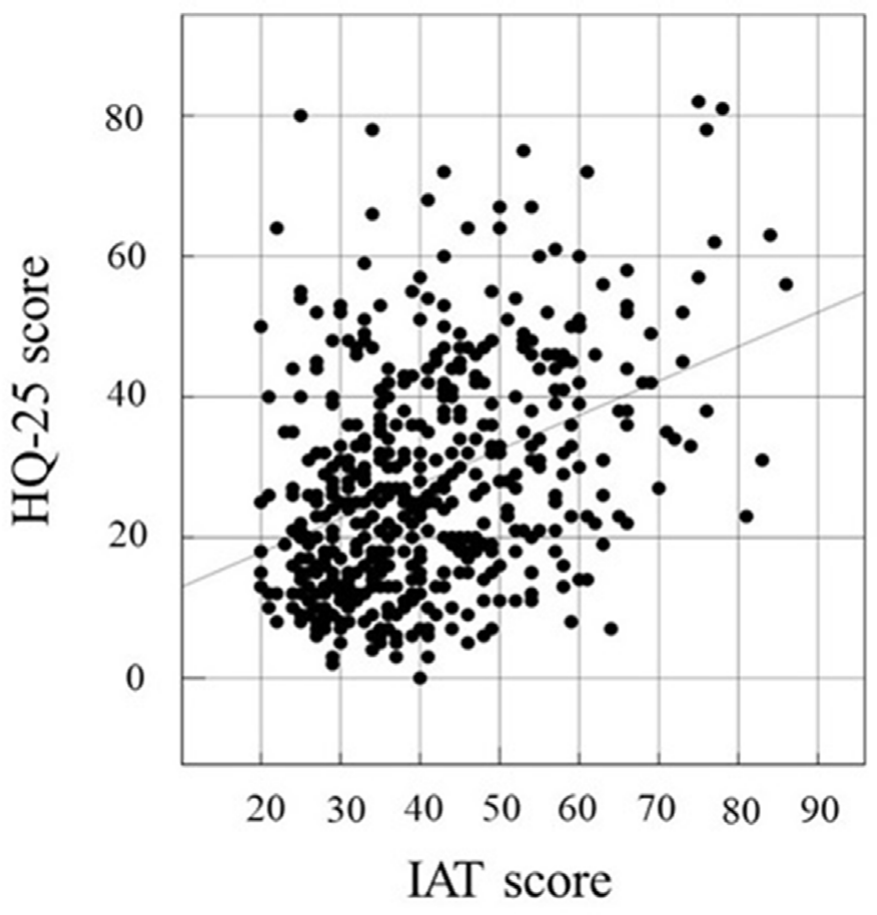
Correlation of IAT (51) score and HQ-25 (52). The correlation coefficient was 0.3905 (95% conﬁdence interval, 0.3125 − 0.4633, p < 0.00001).

We investigated the correlation between two of the following three factors in this study: HQ-25, SAS-SV, and IAT. The association between two factors was examined by calculating Pearson’s correlation coefficient. Correlations between IAT and SAS-SV (**Figure 1**), HQ-25 and SAS-SV (**Figure 2**), and HQ-25 and IAT (**Figure 3**) were analyzed. The correlation coefficient was 0.6931 (95% conﬁdence interval, 0.6439 - 0.7366) (p < 0.00001) for IAT and SAS-SV, suggesting a relatively strong positive correlation. A weak correlation was observed between HQ-25 and IAT (r = 0.3905, 95% conﬁdence interval, 0.3125 - 0.4633, p < 0.00001). However, between HQ-25 and SAS-SV, we could scarcely find a correlation (r = 0.1636, 95% conﬁdence interval, 0.0758 - 0.2488, p = 0.00029).

## Discussion

Internet addiction is characterized by excessive and problematic internet use and clinical features of behavioral addiction: preoccupation, compulsive behavior, lack of control, and functional impairment. Internet addiction has been attracting increasing social interest because the problems related to internet use are becoming more and more serious. Recently, more than half of internet users in Japan access the internet through their smartphones. As the number of smartphone users increases, problems related to smartphone use become more serious too. Neither internet addiction nor smartphone addiction are listed as a psychiatric disorder in the latest version of DSM and ICD. However, since internet addiction is a major public health problem, the Korean government implemented an online gaming shutdown policy in 2011 to prevent internet addiction in adolescents less than 16 years old and established many government-funded centers to support the recovery of the subjects with an internet addiction (66).

Excessive online gaming is the most common cause of internet addiction throughout the world. About one quarter of adolescent males use the internet to enjoy online gaming, according to the report by MIAC (27). Problematic internet use for gaming is a serious concern in many countries and sudden death cases in adolescents during gaming have been reported from several Asian countries. In response to increased awareness of the seriousness of pathological gaming, in the DSM-5, “Internet Gaming Disorder” is included in section III as “Conditions for Further Study,” suggesting the possibility of it being recognized as an independent clinical entity in a future revision. Furthermore, the ICD-11 published in June 2018 included “Gaming Disorder” under the heading of “Disorders due to addictive behaviors.” In ICD-11, gaming disorder is characterized by a pattern of persistent or recurrent gaming behavior, which may be online or offline, manifested by: 1) impaired control over gaming; 2) increasing priority given to gaming to the extent that gaming takes precedence over other life interests and daily activities; and 3) continuation or escalation of gaming despite the occurrence of negative consequences. The pattern of gaming behavior is of sufficient severity to result in significant functional impairment over a period of at least 12 months. Conceptualization of gaming disorder will facilitate future studies on this issue.

Results from our study revealed that users who mainly use the internet for gaming also use the internet significantly longer on both weekdays and weekends and their mean IAT score was significantly higher than those who use the internet for SNSs. It is notable that the mean HQ-25 score in gamers was much higher than SNS users, but no statistically significant difference was found between these two groups on the mean SAS-SV score. A cross-country survey among adolescents reported that males favored playing games on a computer. It is presumed that they prefer online gaming on a computer with a larger screen and a connected controller specially designed for gaming, although they would play games on a smartphone outside of their homes. In general, most of the male gamers enjoy online gaming in their own rooms and some of them enjoy it with their friends while chatting online. Considering these findings, internet users for gaming, mainly male adolescents and youth, are relatively isolated and withdrawn and prefer gaming to social activity.

It is notable that the rates of the subjects who screened positive for hikikomori (22.2%) and smartphone addiction (39.8%) were relatively high despite the study subjects being healthy college/university students. The internet has changed our lives dramatically and has become a daily necessity globally. The mean age of the subjects was 19.6 ± 1.5 years old. Because the internet has become an indispensable tool for our daily lives in the last twenty years, all the subjects of this study have been exposed to advanced IT technology since their early childhood. The internet has altered the way we communicate with each other and asocial individuals might only need to make a minimal effort to communicate with their friends. Nowadays, social interactions tend to occur directly on the internet and the necessity of talking with their friends in person might be decreased. Young generations who have grown up with the internet could prefer to interact with others online rather than engage in interpersonal communication in the real world. Of course, our subjects were essentially not hikikomori cases because they were attending their classes. However, about one fifth of our subjects may potentially be psychologically withdrawn from the social network of their classmates despite not being physically withdrawn from the classroom. Regarding the high rate of positive screenings for smartphone addiction, since most of the youth have their smartphones with them anytime and anywhere, they might already be aware of their overuse. It could be common for them to have social interactions with their friends online through their smartphone.

As the number of the internet users increases, the number of SNS users becomes higher as well. The term “Facebook addiction” has been established and several studies on this theme have been published mainly from European countries (67–69). This concept emerged due to the fact that the number of active Facebook users is outstandingly high compared to other SNS sites (70). In Japan, because the number of LINE users is two-fold higher than Facebook among the youth (1), we need to monitor excessive and problematic use of LINE carefully.

LINE is the most popular SNS application among Japanese youth. It is used for sending text messages and making free calls, mainly on smartphones. Nowadays, people share their LINE ID in their basic contact details. Even in the classroom, at the beginning of the first term, students share their LINE IDs to make semi-official contact networks with classmates to share important information. Some of the enjoyable features of LINE may contribute to its popularity, including simplicity of setting up private and “closed” chat groups with other users, and sending LINE stickers or so-called “stamps,” amusing graphic illustrations and emoticons. Our results showed that about 60% of our subjects chose LINE as their favorite SNS application. This rate of LINE users is consistent with results from a previous large-scale survey (1).

Several factors may inadvertently cause excessive LINE use among adolescent girls. One of them could be a “(has been) Read” sign. A “Read” sign shows up on sender’s smartphone once a message has been read by other group members. LINE users might experience significant distress in this context. Message senders without prompt response from others could perceive they might be ignored or neglected intentionally, and vice versa. Message recipients could be obsessive about checking the screen and replying to the message promptly. Thus, they have their smartphone with them all day long being tied to the internet not to be excluded from the closed chat groups on LINE.

In this study, females scored significantly higher than males on SAS-SV and more than 40% of female subjects scored higher than the proposed cutoff point, suggesting that they might be addicted to their smartphone. Two-group comparisons between Gamers and SNS users showed that the subjects who use the internet for SNS scored significantly lower on HQ-25 and this result might suggest that they were more socially active to maintain the relationship with their friends than those who use the internet for gaming. In general, males favor online gaming while females use the internet primarily for chatting with peers on SNS applications *via* smartphone (25, 26, 71). As we mentioned earlier, young females continue to exchange messages throughout the day on LINE. However, social media applications in the closed online community could be a platform for cyberbullying. Bullying at schools is a serious social problem in Japan (72). In June 2013, a law against bullying, the Ijime Prevention Methods Promotion law, was enacted in Japan, requiring all schools to make efforts to prevent bullying and enable its detection for appropriate intervention at earlier stages. In this law, bullying is defined as “any physical or psychological assault, regardless of location, means or occasion, if the targeted victim suffers psychological distress.” According to this definition, this law clearly includes cyberbullying. Cyberbullying can be triggered by subtle actions, such as someone not replying to a message after reading it. Because the pause after the “(has been) Read” sign could imply various meanings, like neglect or disregard, LINE users will check their smartphones obsessively and compulsively, craving a response. Socially sensitive female adolescents and youth are staying connected to the internet, whether they are enjoying it or not.

We also compared the results of two groups: at high and low risk for hikikomori. According to the cutoff score proposed by the developer of HQ-25, subjects who scored 42 and higher were grouped as high risk for hikikomori while those who scored less than 42 were treated as low risk for hikikomori group. Subjects in high risk for hikikomori group used internet much longer and scored significantly higher on both SAS-SV and IAT than those in low risk for hikikomori group. It has been reported that hikikomori phenomena are related to other psychiatric disorders, including developmental disorders (28, 31, 33, 34, 37). Internet addiction often has comorbid psychiatric and developmental disorders (73–77). It is possible that internet addiction and hikikomori have similar psychopathological backgrounds. Autism spectrum disorder (ASD) is characterized by two core features: impairments in social interaction and restricted, repetitive behavior. Attention deficit hyperactivity disorder (ADHD) is defined by three symptoms: inattention, hyperactivity, and impulsivity. Young initially proposed provisional diagnostic criteria for subjects with problematic internet use with her naming of “Internet addiction” in reference to DSM-5-TR criteria for substance dependence (51). After lively debate on conceptualizing problematic internet use as one type of behavioral addictions, Young revised her definition of IA and made it closer to an Impulse Control Disorder (8). Impulsivity has been associated with addictive behaviors in adolescents (78). Both ASD and ADHD often exist as comorbid conditions (79–81). Clinical features of these common developmental disorders, i.e., restricted and repetitive type of behavior and difficulty in controlling impulsivity, could contribute to internet addiction. Impairments in social interaction would be an important factor of hikikomori. Disconnection from relationships with friends as a result of pathological internet use will keep the subjects connected to the internet instead.

The argument that internet addiction could be a cause of hikikomori often ends up in a chicken or egg situation. However, there is no room for discussion about a fact that a large number of hikikomori subjects are online almost all through the day in their own rooms or houses. Not all but some youths with hikikomori traits are socially active only in virtual spaces on the internet as an anonymous internet user or by acting a different personality through an avatar, the graphical representation of the internet user. Many male internet users enjoy playing online games with “internet friends” without disclosing their personal information such as age, gender, place to live and name. They are connected with many “internet friends” while being online, but they could be socially isolated to some extent in their real lives. As for female internet users, they remain being online *via* smartphones so as not to be excluded from their communications on SNS applications such as LINE and seem to be socially active on the internet superficially. However, some of female SNS users are overwhelmed by a large number of messages and might be afraid of opening the social media application. Female internet users enjoy chattering *via* smartphones, but it is not uncommon for them to have an aversion to face-to-face communications with their friends in the real life. Some young females who are active in social interaction on SNS application often decline an invitation to a party or hesitate to go out for shipping with friends. They might be concerned about being humiliated by making a perceived mistake in their face-to-face conversation and tend to regard it as irretrievable. Youths could be traumatized more seriously by an undesirable event in the real life compared to what happened on the internet.

Recently, the concept of Modern-Type Depression (MTD) has been proposed and is getting more attention in Japan (82). Characteristics of MTD include situation-dependent depressive symptoms, a disposition to blame others, and strong avoidant tendencies including social avoidance (83). Youths with MTD could exhibit absenteeism from their work and spend much time on the internet. MTD has something in common with hikikomori in terms of avoiding social situations and could be a gateway condition that leads to hikikomori.

There are several limitations in this study. The sample size was limited, and only college and university students were invited to participate. It is impossible to generalize our results because of a significant sampling bias, including the lopsided male-female ratio of the study subjects. None of our subjects underwent a clinical interview. We did not use multiple measures to assess hikikomori condition. Loneliness and social isolation were not assessed in this study, although both of them are similar to hikikomori. It is difficult to distinguish internet addiction from excessive internet use precisely using self-rating scales. The scales used in this study, such as the SAS-SV, IAT, and HQ-25, have limited validity. We also did not assess for any formal diagnosis of psychiatric disorders in our subjects. The subjects at high risk for hikikomori group in this study were not in the essential hikikomori condition in its original meaning because they attended the classroom. Because this is not a longitudinal study but a cross-sectional study, we cannot confirm the stability of relationship among the variables assessed in this study.

To the best of our knowledge, this is the first study to investigate the relation among internet addiction, smartphone addiction and risk for hikikomori in Japanese young adults. As the number of internet and smartphone users increases, the problem of internet/smartphone addiction will become more prevalent in Japan, and one of possible sequelae of these conditions will be hikikomori. Mental health providers should be aware of the seriousness of behavioral addictions and hikikomori. Risk factors of hikikomori include internet addiction and instructions for appropriate internet use could be a protective factor. We envision that further studies will invite a larger number of subjects with a wider age range, as well as include a clinical interview to confirm the condition of internet/smartphone addiction and hikikomori.

## Ethics Statement

The study protocol was approved by the ethics committee of Tokiwa Hospital prior to data collection. This study was conducted in accordance with the Declaration of Helsinki. The aim of this study was clearly stated on the first page of the anonymous questionnaire sheets. Response to the questionnaire was deemed indicative of consent.

## Author Contributions

MT contributed to the study design, data collection, data analysis, data interpretation and writing. AT contributed to the study design, data interpretation, and writing. WU and JK contributed to the data collection and data interpretation. RK and HK contributed to the data analysis and data interpretation. TK contributed to the study design, data interpretation, and writing.

## Funding

This work was partially supported by a Grant-in-Aid for Scientific Research from 1) The Japan Agency for Medical Research and Development (AMED) (Syogaisya-Taisaku-Sogo-Kenkyu-Kaihatsu-Jigyo; JP19dk0307073h to MT and TK) and 2) Innovative Areas “Will-Dynamics” of The Ministry of Education, Culture, Sports, Science, and Technology, Japan (JP16H06403 to TK).

## Conflicts of Interest Statement

The authors declare that the research was conducted in the absence of any commercial or financial relationships that could be construed as a potential conflict of interest.
